# Viability of *Dugesia dorotocephala* Planaria with the Senolytic Drug ARV-825

**DOI:** 10.17912/micropub.biology.001636

**Published:** 2026-02-06

**Authors:** Abbie Wells, William H. Munroe

**Affiliations:** 1 California State University, Channel Islands, Camarillo, California, United States

## Abstract

Planaria ( *Dugesia dorotocephala* ) are being utilized as a model system to test the effectiveness of ARV-825. ARV-825 belongs to the drug class proteolysis targeting chimeras (PROTACs), which targets and degrades the protein Bromodomain-containing protein 4 (BRD4) via the proteasome. To examine the impact of ARV-825 on the planaria, planaria viability was tested at different ARV-825 concentrations. This demonstrated a concentration-dependent reduction in viability, 91.7% survival with 1µM ARV-825 reduced to 25% survival in 10 µM ARV-825. This suggests ARV-825 has an effect on *Dugesia dorotocephala* , and indicates that this may be a suitable model organism for studying ARV-825.

**Figure 1. Study data investigating the viability of Dugesia dorotocephala planaria with the senolytic drug ARV-825:**
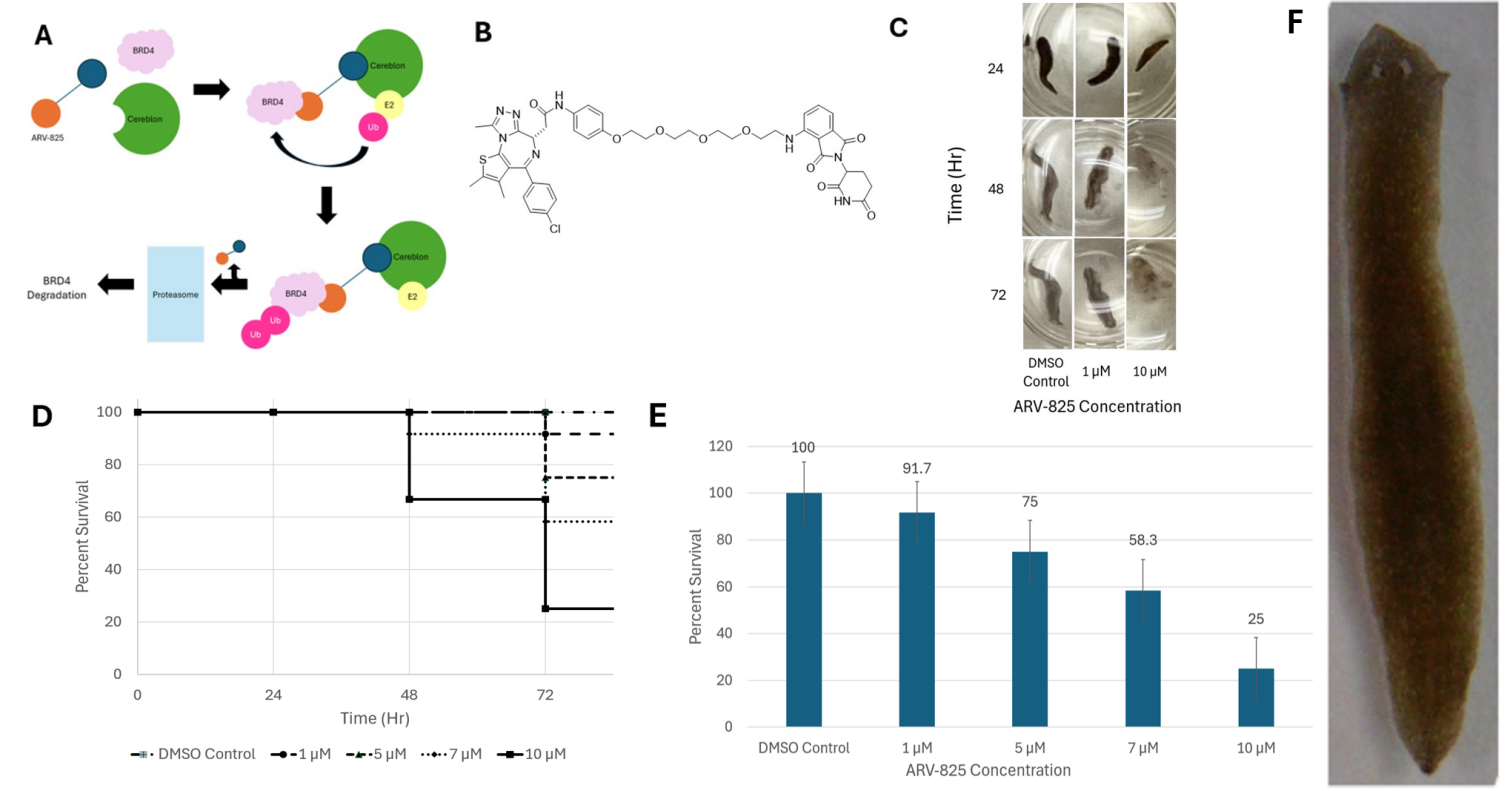
&nbsp; **A)** ARV-825-mediated degradation mechanism for BRD4. When introduced, ARV-825 binds to the BRD4 protein complex and the E3 Ligase Substrate Adapter Cereblon. Once the ubiquitin (Ub) is attached to the BRD4 protein, successfully marking it for degradation, the ARV-825 detaches from the BRD4 and the E3 Ligase Substrate Adapter Cereblon. The ubiquitinated BRD4 protein then moves through the proteasome, where it is degraded, (Liao et al., 2021). **B)** The molecular structure of ARV-825, IUPAC name 2-((S)-4-(4-chlorophenyl)-2,3,9-trimethyl-6H-thieno[3,2-f][1,2,4]triazolo[4,3-a][1,4]diazepin-6-yl)-N-(4-(2-(2-(2-(2-((2-(2,6-dioxopiperidin-3-yl)-1,3-dioxoisoindolin-4yl)amino)ethoxy)ethoxy)ethoxy)ethoxy)phenyl)acetamide. **C)** Worms incubated in 0 µM, 1 µM, and 10 µM ARV-825 were photographed at 24, 48, and 72 hours. Worms were imaged using a cellphone camera under ambient lighting conditions. **D)** Planaria survival throughout a 72-hour incubation. This data is the percent survival of the worms incubated with various concentrations of ARV-825. The data was collected over three days at 24-hour intervals and was conducted in triplicate, with 4 worms per replicate (n=4), for a total of 12 worms (n=12) per concentration tested.&nbsp; Worms were scored as dead in the event of autolysis or if the worm exhibited a complete lack of movement when prodded. **E)** Final planaria survival after 72-hour incubation. Only data collected at 72 hours is presented. Error bars represent the standard error of the mean of the dataset. **F)** *Dugesia dorotocephala* as supplied from Carolina Biological (Brown Planaria). Worms were imaged using a TOMLOV DM401 digital microscope with LED illumination.

## Description

As individuals age, they face an increased risk of chronic diseases and a range of geriatric syndromes (Kirkland and Tchkonia, 2020; Wissler Gerdes et al., 2020; Kirkland et al., 2016; Kirkland, 2013a, 2016b). Aging is a major risk factor for conditions such as cardiovascular diseases, dementia, cancer, diabetes, and metabolic disorders. Complications in the lungs, kidneys, bones, and joints are also significant risk factors associated with aging (Kirkland and Tchkonia, 2020a, 2017b; Lewis-Mcdougall et al., 2019; Kennedy et al., 2014; Kirkland et al., 2002). These health conditions are a strong research topic as they are difficult to prevent and treat (Kirkland and Tchkonia, 2020; Wissler Gerdes et al., 2020; Kirkland et al., 2016; Kirkland, 2013a, 2016b). In tandem with chronic conditions, aging is linked to a decreased recovery rate from natural stressors such as cardiovascular events, dehydration, and infections. Reduced recovery rates are also observed in patients undergoing medical interventions like chemotherapy or surgery (Kirkland and Tchkonia, 2020; Wissler Gerdes et al., 2020; Kirkland et al., 2016; Kirkland, 2013a, 2016b). This strongly suggests that the aging process itself may be the root cause of these conditions (Wissler Gerdes et al., 2020; Kennedy et al., 2014). This aging process may also be influenced by cellular senescence.&nbsp;

Cellular senescence produces a near-irreversible cessation of replication with corresponding changes to apoptosis resistance and increased metabolic activity (Kirkland and Tchkonia, 2020; Wissler Gerdes et al., 2020; Kirkland et al., 2016; Kirkland, 2013a, 2016b; Kennedy et al., 2014). While these cells have limited reproduction, they are still metabolically active (Wissler Gerdes et al., 2020; Kennedy et al., 2014). The intra- and extracellular signals linked to cells becoming senescent include signals associated with tissue damage, cellular damage, and cancer development (Kirkland and Tchkonia, 2020; Wissler Gerdes et al., 2020; Kirkland et al., 2016; Kirkland, 2013a, 2016b). Similar to cancer cells, senescent cells are commonly shifted from fatty acid utilization to glycolysis. This metabolic change not only supports the senolytic cell’s survival but also leads to many dysfunctional consequences, such as an increase in reactive oxygen species (ROS) generation. This ROS increase contributes to oxidative stress and cellular damage. Additionally, it promotes lipid accumulation within the cells, overwhelming their metabolic capacity and resulting in lipotoxicity (Wissler Gerdes et al., 2020; Kirkland et al., 2002).&nbsp;

In tandem, a high concentration of senescent cells in the body can lead to local and systemic inflammation, tissue damage, and immune system suppression. This is due to senescence-associated secretory phenotypes (SASPs) (Wissler Gerdes et al., 2020; Kirkland, 2013; Kirkland et al., 2002). The SASP that develops depends on the type of senescent cell and the underlying cause of senescence. Senescent cells attract and activate immune cells, allowing the immune cells to become anchored to the senescent cells. If left untreated, a buildup of senescent cells can disrupt normal tissue function by triggering fibrosis, damaging DNA, impairing mitochondria, and contributing to harmful protein aggregation (Wissler Gerdes et al., 2020; Kirkland et al., 2002).&nbsp;

*Dugesia dorotocephala* is a freshwater planarian species native to North America and has been widely used as an experimental model for studying regeneration and stem-cell biology (Almazan et al., 2018). *D. dorotocephala* exhibits tissue functions shared across metazoans, making it a powerful model system for studying the fundamental biological processes of tissue maintenance and repair (Reddien & Sánchez Alvarado, 2004; Elliott & Sánchez Alvarado, 2013). Its regenerative capacity is driven by a population of adult pluripotent stem cells known as neoblasts, which are capable of giving rise to all differentiated cell types required for regeneration (Reddien et al., 2005; Wagner et al., 2011). Following injury or amputation, neoblasts proliferate and migrate to the wound site to form a blastema, enabling complete and correctly patterned tissue restoration. Due to its experimental tractability, low maintenance cost, and regenerative response, *D. dorotocephala* remains an important model for studying stem-cell regulation and regeneration mechanisms (Elliott & Sánchez Alvarado, 2013).

Beyond regeneration biology, planarians have a long and significant history in pharmacological, toxicological, and neurobiological studies. Early studies used planarians as models in behavior, neuropharmacology, and drug screening studies, as their simple yet functionally complex nervous system exhibits drug responses comparable to those observed in humans (Pagán, 2014; Pagán, 2017). More recently, planarians have been proven valuable alternatives for studying developmental neurotoxicity, brain regeneration, and aging (Hagstrom et al., 2016; Wu and Li, 2018; Holtze et al., 2021; Collins et al., 2024).&nbsp;

The combination of planarians’ high sensitivity to environmental and chemical exposures, their conserved neurotransmitter systems, and quantifiable behavioral responses makes them an effective model for toxicology and pharmacology research (Holtze et al., 2021; Pagán, 2017; Wu and Li, 2018). Additionally, recent reviews state that planarians continue to serve as a paradigm-shifting model in regeneration biology (Elliott and Alvarado, 2018; Newmark and Sánchez Alvarado, 2022). Their regenerative capacity not only highlights the fundamental principles of stem cell biology but also makes them a powerful model system for predicting pharmacological and toxicological effects (Hagstrom et al., 2016; Holtze et al., 2021; Pagán, 2017; Wu and Li, 2018).

Neuroblastoma (NB) is a common solid childhood tumor. Previously, the inhibition of bromodomain and extra-terminal (BET) protein showed potential in treating these tumors (Kennedy et al., 2014). ARV-825, IUPAC name (2-((S)-4-(4-chlorophenyl)-2,3,9-trimethyl-6H- thieno[3,2-f][1,2,4]triazolo[4,3-a][1,4]diazepin-6-yl)-N-(4-(2-(2-(2-(2-((2-(2,6-dioxopiperidin-3-yl)-,3-dioxoisoindolin-4yl)amino)ethoxy)ethoxy)ethoxy) ethoxy)phenyl)acetamide) is a novel senolytic BET inhibitor that uses proteolysis-targeting chimera technology. This targets proteins for degradation via the proteasome (Li et al., 2020). When introduced, ARV-825 binds to the BRD4 protein complex and the E3 Ligase Substrate Adapter Cereblon. Once the ubiquitin is attached to the C-terminal domain of the BRD4 protein, successfully marking it for degradation, the ARV-825 detaches from the BRD4 and the E3 Ligase Substrate Adapter Cereblon. The ubiquitinated BRD4 protein then moves through the proteasome, where it is degraded (Liao et al., 2021). Manipulation of modifiers, like inhibiting the bromodomain and proteins that link chromatin markers to activate transcription, has also been proven to be an effective way to block cell expression (Li et al., 2020).&nbsp;

The BET family, which includes BRD2, BRD3, BRD4, and BRDT, can recognize and bind acetylated lysine modifications of histones, which facilitates chromatin remodeling and transcriptional activation (Li et al., 2020). This activity is particularly important in cancer and inflammation, making BET proteins a therapeutic target. ARV-825 specifically degrades BET proteins to suppress tumor growth and modify transcription (Li et al., 2020). BRD4 has been shown to promote resistance to apoptosis in cancer cells by sustaining the expression of pro-survival genes (Zuber et al, 2011). Therefore, targeted degradation of BRD4 by ARV-825 may enhance apoptotic sensitivity, making it an attractive strategy for eliminating tumor cells. This experiment examines the effect of ARV-825, a BET inhibitor, on planaria ( *Dugesia dorotocephala* ) to evaluate its potential in senolytic treatment by promoting apoptosis in senescent cells.

Dimethyl sulfoxide (DMSO) is a widely used solvent in toxicology and pharmacology studies involving planarians. However, multiple reports indicate that it can independently cause significant behavioral effects. For example, exposure to 0.1–3% DMSO causes reversible, dose-dependent reductions in motility and antioxidant enzyme activity, with full recovery only at the lower end of this range. (Yuan et al., 2012). Similarly, non-lethal concentrations of DMSO impair motility and other behavioral responses (Stevens et al., 2015). Even at concentrations considered nontoxic, behavioral changes such as reduced locomotor activity and altered phototaxis have been seen (Pagán et al., 2006; Pagán et al., 2009). While 0.9% DMSO used in this study is generally regarded as nonlethal in planarians, earlier studies show it can still affect behavior in ways that might interact with or enhance the apparent toxicity of additional treatments (Yuan et al., 2012; Stevens et al., 2015; Pagán et al., 2006). Thus, 0.9% DMSO was employed to satisfy the solubility requirements of ARV-825, and because prior studies indicate that planarians generally tolerate concentrations below 1–2% without causing irreversible effects (Yuan et al., 2012; Stevens et al., 2015).

Figure 1A denotes the ARV-825-mediated degradation mechanism for BRD4. When introduced, ARV-825 binds to the BRD4 protein complex and the E3 Ligase Substrate Adapter Cereblon. Once the ubiquitin (Ub) is attached to the BRD4 protein, successfully marking it for degradation, the ARV-825 detaches from the BRD4 and the E3 Ligase Substrate Adapter Cereblon. The ubiquitinated BRD4 protein then moves through the proteasome, where it is degraded. Figure 1B denotes the chemical structure of ARV-825.&nbsp;

Figure 1C shows photos of worms treated with 0 µM, 1 µM, and 10 µM ARV-825 at 24, 48, and 72 hours. DMSO control appears to experience little to no change, while the 1 µM and 10 µM ARV-825 samples exhibit visible changes. Beginning at 48 hours for both 1 µM and 10 µM ARV-825 samples, the worms became more “blurry” or “transparent,” possibly the result of various apoptosis events causing tissue death. The 1 µM ARV-825-treated worms experienced significantly less of the “blurry” phenotype over time when compared to the 10 µM ARV-825-treated worms. By the conclusion of the 72-hour incubation, the 10 µM ARV-825-treated worm in Figure 1B only retained approximately 60% of its body while maintaining the ability to move throughout the well. As the DMSO Control worms showed no change throughout the incubation, these changes may be due to apoptosis events attributed to ARV-825.&nbsp;

Figure 1D highlights the overall planaria survival at each concentration of ARV-825, ranging from 1 µM to 10 µM. The data was collected at 0, 24, 48, and 72-hour intervals. Increasing concentrations of ARV-825 resulted in higher mortality, as a 100% survival rate was observed in the DMSO control, 91.7% survival with 1µM ARV-825, 75.0% survival in 5 µM ARV-825, 58.3% survival in 7µM ARV-825, and 25.0% survival in 10 µM ARV-825 was observed. Looking at Figure 1E, which shows the final planaria survival after the 72-hour incubation, it is seen that the DMSO control worms had the highest survival rate, in which all of the worms survived, and 10 µM had the lowest survival rate, in which only 25.0% of the worms survived. Since all samples contained the same concentration of DMSO and no mortality was observed in the DMSO control worms, DMSO was not a contributing factor in worm death. As the drug concentration and time increased, the worm mortality increased, showing the potency of the drug. The error bars in Figure 1E represent the standard error of the mean of the data set. Both Figures 1D and 1E were performed as a triplicate, with 4 worms per replicate (n=4), for a total of 12 worms (n=12) per concentration tested. Finally, Figure 1F depicts the *Dugesia dorotocephala* worms as supplied from Carolina Biological (Brown Planaria). Worms were imaged using a TOMLOV DM401 digital microscope with LED illumination.

This data provides valuable insights into the planaria *Dugesia dorotocephala* ’s mortality in ARV-825. There was a great difference in the final mortality of the lowest (1 µM) and highest concentration (10 µM) incubations: 91.7% vs 25.0%, respectively. The blurry phenotype rates also varied between the concentrations, with 10 µM samples having the highest level of observed visual degradation to the worm body. This may be due to drug-induced apoptosis in several regions of the worm.&nbsp;

Future studies should determine the mechanism of action underlying ARV-825-induced mortality in *Dugesia dorotocephala* planaria. These studies will focus on identifying whether apoptosis or other stress pathways are responsible for the observed degradation in Figure 1B . Clarifying the drug’s mode of action will help determine its therapeutic specificity, efficiency, and potential.

## Methods

*Planaria Housing and Care:* The worms (Brown Planaria) were obtained from Carolina Biological and were housed in containers consisting of Instant Ocean mix: 0.5 g of Instant Ocean salt per 1 L of deionized water (King and Newmark, 2018). Worms were fed blended calf liver paste for at least one hour (King and Newmark, 2018). After feeding, the water was changed to remove excess liver paste. Worms were stored in a dark, minimally disturbed place and fed at least once per week until used. They were housed in these conditions for a minimum of 2 weeks before drug incubation.

*Drug Incubation:* ARV-825 powder was dissolved in DMSO to create a stock of 108 µM. Using the 0.5g/L Instant Ocean mix, the ARV-825 was administered at the following concentrations: 1.0µM, 5.0µM, 7µM, and 10µM. All samples were adjusted to have the same final percentage of DMSO, 0.9% (v/v). Using a 24-well plate, the worms were incubated in 1 mL of solution for 72 hours in a dark, undisturbed place, with data taken every 24 hours. The experiment was conducted in triplicate, with 4 worms per replicate (n=4), for a total of 12 worms (n=12) per concentration tested. Worms were scored as dead in the event of autolysis or if the worm exhibited a complete lack of movement when prodded.

## References

[R1] Almazan EMP, Lesko SL, Markey MP, Rouhana L. 2018. Girardia dorotocephala transcriptome sequence, assembly, and validation through characterization of piwi homologs and stem cell progeny markers. Dev Biol. 433(2):433-447. doi: 10.1016/j.ydbio.2017.07.022. PMID: 28774726; PMCID: PMC5750089 10.1016/j.ydbio.2017.07.022 PMC5750089 28774726

[R2] Collins, E.-M.S., Hessel, E.V.S., Hughes, S., 2024. How neurobehavior and brain development in alternative whole-organism models can contribute to prediction of developmental neurotoxicity. NeuroToxicology 102, 48–57. https://doi.org/10.1016/j.neuro.2024.03.005 10.1016/j.neuro.2024.03.005 PMC11139590 38552718

[R3] Elliott SA, Sánchez Alvarado A. 2012. The history and enduring contributions of planarians to the study of animal regeneration. Wiley Interdiscip Rev Dev Biol. 2(3):301-26. doi: 10.1002/wdev.82. Epub 2012 Jul 23. PMID: 23799578; PMCID: PMC3694279 10.1002/wdev.82 PMC3694279 23799578

[R4] Elliott, S.A., Alvarado, A.S., 2018. Planarians and the History of Animal Regeneration: Paradigm Shifts and Key Concepts in Biology, in: Rink, J.C. (Ed.), Planarian Regeneration. Springer New York, New York, NY, pp. 207–239. https://doi.org/10.1007/978-1-4939-7802-1_4

[R5] Hagstrom, D., Cochet‐Escartin, O., Collins, E.S., 2016. Planarian brain regeneration as a model system for developmental neurotoxicology. Regeneration 3, 65–77. https://doi.org/10.1002/reg2.52 10.1002/reg2.52 PMC4895328 27499880

[R6] Holtze, S., Gorshkova, E., Braude, S., Cellerino, A., Dammann, P., Hildebrandt, T.B., Hoeflich, A., Hoffmann, S., Koch, P., Terzibasi Tozzini, E., Skulachev, M., Skulachev, V.P., Sahm, A., 2021. Alternative Animal Models of Aging Research. Front. Mol. Biosci. 8, 660959. https://doi.org/10.3389/fmolb.2021.660959 10.3389/fmolb.2021.660959 PMC8166319 34079817

[R7] Kennedy BK, Berger SL, Brunet A, Campisi J, Cuervo AM, Epel ES, Francesch C, Lithgow G, Morimoto RI, Pessin JE, et al. 2014. Geroscience: linking aging to chronic disease. Cell. 159(4):709–713. https://doi.org/10.1016/j.cell.2014.10.039 10.1016/j.cell.2014.10.039 PMC4852871 25417146

[R8] King RS, Newmark PA. 2018. Whole-mount in situ hybridization of planarians. Mol. Biol. 1774:379–392. https://doi.org/10.1007/978-1-4939-7802-1_12 10.1007/978-1-4939-7802-1_12 29916165

[R9] Kirkland JL. 2013. Translating advances from the basic biology of aging into clinical application. Exp. Gerontol. 48(1):1–5. https://doi.org/10.1016/j.exger.2012.11.014 10.1016/j.exger.2012.11.014 PMC3543864 23237984

[R10] Kirkland JL. 2016. Translating the Science of Aging into Therapeutic Interventions. Cold Spring Harb. Perspect. Med. 6(3):a025908. https://doi.org/10.1101/cshperspect.a025908 10.1101/cshperspect.a025908 PMC4772076 26931808

[R11] Kirkland JL, Stout MB, Sierra F. 2016. Resilience in Aging Mice. J. Gerontol. A Biol. Sci. Med. Sci. 71(11):1407–1414. https://doi.org/10.1093/gerona/glw086 10.1093/gerona/glw086 PMC5865545 27535963

[R12] Kirkland JL, Tchkonia T, Pirtskhalava T, Han J, Karagiannides I. 2002. Adipogenesis and aging: does aging make fat go MAD?. Exp. Gerontol. 37(6):757–767. https://doi.org/10.1016/s0531-5565(02)00014-1 10.1016/s0531-5565(02)00014-1 12175476

[R13] Kirkland JL, Tchkonia T. 2017. Cellular Senescence: A Translational Perspective. EBioMedicine. 21:21–28. https://doi.org/10.1016/j.ebiom.2017.04.013 10.1016/j.ebiom.2017.04.013 PMC5514381 28416161

[R14] Kirkland JL, Tchkonia T. 2020. Senolytic drugs: from discovery to translation. J. Intern. Med. 288(5):518–536. https://doi.org/10.1111/joim.13141 10.1111/joim.13141 PMC7405395 32686219

[R15] Lewis-McDougall FC, Ruchaya PJ, Domenjo-Vila E, Shin Teoh T, Prata L, Cottle BJ, Clark JE, Punjabi P, Awad W, Torella D, et al. 2019. Aged-senescent cells contribute to impaired heart regeneration. Aging cell. 18(3):e12931. https://doi.org/10.1111/acel.12931 10.1111/acel.12931 PMC6516154 30854802

[R16] Li Z, Lim SL, Tao Y, Li X, Xie Y, Yang C, Zhang Z, Jiang Y,&nbsp; Zhang X, Cao X, et al. 2020. PROTAC Bromodomain Inhibitor ARV-825 Displays Anti-Tumor Activity in Neuroblastoma by Repressing Expression of MYCN or c-Myc. Front Oncol. 10:574525. https://doi.org/10.3389/fonc.2020.574525 10.3389/fonc.2020.574525 PMC7726414 33324552

[R17] Liao X, Qian X, Zhang Z, Tao Y, Li Z, Zhang Q, Liang H, Li X, Xie Y, Zhuo R, et al. 2021. ARV-825 Demonstrates Antitumor Activity in Gastric Cancer via MYC-Targets and G2M-Checkpoint Signaling Pathways. Front Oncol. 11:753119. https://doi.org/10.3389/fonc.2021.753119 10.3389/fonc.2021.753119 PMC8559897 34733788

[R18] Newmark, P.A., Sánchez Alvarado, A., 2022. *Schmidtea * happens: Re-establishing the planarian as a model for studying the mechanisms of regeneration, Curr. Top. Dev. Biol. Elsevier, pp. 307–344. https://doi.org/10.1016/bs.ctdb.2022.01.002 10.1016/bs.ctdb.2022.01.002 35337453

[R19] Pagán OR, Coudron T, Kaneria T. 2009. The flatworm planaria as a toxicology and behavioral pharmacology animal model in undergraduate research experiences. J Undergrad Neurosci Educ. 7(2):A48-52 PMC3592692 23493443

[R20] Pagán OR, Rowlands AL, Urban KR. 2006. Toxicity and behavioral effects of dimethylsulfoxide in planaria. Neurosci Lett. 30;407(3):274-8. 10.1016/j.neulet.2006.08.073 10.1016/j.neulet.2006.08.073 16979295

[R21] Pagán, O.R., 2014. The first brain: the neuroscience of planarians. Oxford University Press, Oxford (GB)

[R22] Pagán, O.R., 2017. Planaria: an animal model that integrates development, regeneration and pharmacology. Int. J. Dev. Biol. 61, 519–529. https://doi.org/10.1387/ijdb.160328op 10.1387/ijdb.160328op 29139537

[R23] Reddien PW, Bermange AL, Murfitt KJ, Jennings JR, Sánchez Alvarado A. 2005 Identification of genes needed for regeneration, stem cell function, and tissue homeostasis by systematic gene perturbation in planaria. Dev Cell. ;8(5):635-49. doi: 10.1016/j.devcel.2005.02.014. PMID: 15866156; PMCID: PMC2267917 10.1016/j.devcel.2005.02.014 PMC2267917 15866156

[R24] Reddien PW, Sánchez Alvarado A. 2004. Fundamentals of planarian regeneration. Annu Rev Cell Dev Biol. 2004;20:725-57. 10.1146/annurev.cellbio.20.010403.095114. PMID: 15473858 10.1146/annurev.cellbio.20.010403.095114 15473858

[R25] Stevens AS, Pirotte N, Plusquin M, Willems M, Neyens T, Artois T, Smeets K. 2015. Toxicity profiles and solvent-toxicant interference in the planarian Schmidtea mediterranea after dimethylsulfoxide (DMSO) exposure. J Appl Toxicol. 35(3):319-26. https://analyticalsciencejournals.onlinelibrary.wiley.com/doi/10.1002/jat.3011 10.1002/jat.3011 24964768

[R26] Wagner DE, Wang IE, Reddien PW. 2011. Clonogenic neoblasts are pluripotent adult stem cells that underlie planarian regeneration. Science.v332(6031):811-6. doi: 10.1126/science.1203983. PMID: 21566185; PMCID: PMC3338249 10.1126/science.1203983 PMC3338249 21566185

[R27] Wissler Gerdes EO, Zhu Y, Tchkonia T, Kirkland JL. 2020. Discovery, development, and future application of senolytics: theories and predictions.FEBS J. 287(12):2418–2427. https://doi.org/10.1111/febs.15264 10.1111/febs.15264 PMC7302972 32112672

[R28] Wu, J.-P., Li, M.-H., 2018. The use of freshwater planarians in environmental toxicology studies: Advantages and potential. Ecotoxicol. Environ. Saf. 161, 45–56. https://doi.org/10.1016/j.ecoenv.2018.05.057 10.1016/j.ecoenv.2018.05.057 29859407

[R29] Yuan Z, Zhao B, Zhang Y. 2012. Effects of dimethylsulfoxide on behavior and antioxidant enzymes response of planarian Dugesia japonica.Toxicol Ind Health. 28(5):449-57. https://journals.sagepub.com/doi/10.1177/0748233711414609 10.1177/0748233711414609 21976142

[R30] Zuber J, Shi J, Wang E, Rappaport AR, Herrmann H, Sison EA, Magoon D, Qi J, Blatt K, Wunderlich M, et al. 2011. RNAi screen identifies Brd4 as a therapeutic target in acute myeloid leukaemia. Nature. 478(7370):524–528. https://doi.org/10.1038/nature10334 10.1038/nature10334 PMC3328300 21814200

